# Multidisciplinary Treatment for Advanced Pancreatic Adenocarcinoma Arising from Esophageal Heterotopic Pancreas in a Young Adult: A Case Report

**DOI:** 10.70352/scrj.cr.26-0088

**Published:** 2026-06-27

**Authors:** Yohei Mizusawa, Kazuhiro Noma, Tomoyoshi Kunitomo, Yasushige Takeda, Hijiri Matsumoto, Masashi Hashimoto, Naoaki Maeda, Shunsuke Tanabe, Masaaki Akai, Shoji Takagi, Tomohiro Toji, Toshiyoshi Fujiwara

**Affiliations:** 1 Department of Gastroenterological Surgery, Okayama University Graduate School of Medicine, Dentistry and Pharmaceutical Sciences, Okayama, Okayama, Japan; 2 Department of Digestive Surgery, Japanese Red Cross Okayama Hospital, Okayama, Okayama, Japan; 3 Department of Pathology, Japanese Red Cross Okayama Hospital, Okayama, Okayama, Japan

**Keywords:** heterotopic pancreas, esophageal neoplasms, adenocarcinoma, neoplastic transformation, robotic-assisted esophagectomy, multidisciplinary care

## Abstract

**INTRODUCTION:**

Heterotopic pancreas (HP) is a congenital anomaly characterized by pancreatic tissue located outside its normal anatomical site, without anatomical or vascular continuity with the pancreas proper. HP typically occurs in the stomach or duodenum, whereas esophageal HP is extremely rare. Malignant transformation is even rarer, with only a limited number of reported cases. We report a case of adenocarcinoma arising from esophageal HP successfully managed by multidisciplinary treatment.

**CASE PRESENTATION:**

A 32-year-old woman presented with persistent epigastric pain accompanied by severe right-sided posterior thoracic pain that was markedly positional, preventing her from resting in the supine position. Upper gastrointestinal endoscopy revealed ectopic gastric mucosa with a papillary elevation in the lower thoracic esophagus (Lt), and endoscopic US (EUS) demonstrated a homogeneous hypoechoic submucosal mass. Contrast-enhanced CT and esophagography identified a well-defined 30-mm lesion arising from the right wall of the Lt, without distant metastasis or lymphadenopathy. Although EUS-guided fine-needle aspiration (EUS-FNA) and referral for a second opinion were recommended, the patient declined both because of intractable pain and requested immediate surgical management. Thoracoscopic tumor resection was therefore performed for diagnostic and therapeutic purposes. Histopathology confirmed pancreatic-type adenocarcinoma arising from Heinrich type I HP, with lymphatic invasion and a positive resection margin. Accordingly, 3 cycles of adjuvant chemotherapy with gemcitabine (1000 mg/m^2^) and nab-paclitaxel (125 mg/m^2^) were administered over 3 months. Follow-up CT and PET-CT showed no recurrence or metastasis, and robot-assisted subtotal esophagectomy was performed 7 months after the initial surgery. Postoperative pathology demonstrated fibrotic scar tissue without residual malignancy. The patient declined further adjuvant therapy and remains free of recurrence 12 months after the second surgery.

**CONCLUSIONS:**

Esophageal HP carcinoma is exceedingly rare. Multidisciplinary treatment following pancreatic cancer protocols enabled curative resection and favorable outcomes. Given the malignant potential, meticulous preoperative evaluation and individualized therapeutic planning are essential.

## Abbreviations

EGJ
: esophagogastric junction
EUS
: endoscopic US
EUS-FNA
: endoscopic US-guided fine-needle aspiration
GnP
: gemcitabine plus nab-paclitaxel
HP
: heterotopic pancreas
IPMN
: intraductal papillary mucinous neoplasm
Lt
: lower thoracic esophagus
Mt
: middle thoracic esophagus
NR
: not reported
PanIN
: pancreatic intraepithelial neoplasia

## INTRODUCTION

HP is pancreatic tissue located outside the normal pancreas without anatomical or vascular continuity.^1,2)^ It most often occurs in the stomach or duodenum; occurrence in the esophagus is extremely rare.^3)^ Many HPs are asymptomatic and are found incidentally on endoscopy or imaging.^4)^ Reported prevalence in autopsy series ranges from about 0.5% to 13.7%.^5)^ Malignant transformation of HP is even rarer,^6)^ and cancers arising from esophageal HP have been reported in 6 cases (**Table 1**).^7–12)^ Preoperative diagnosis is challenging because imaging and endoscopic findings are often nonspecific. EUS-FNA can help, but its sensitivity is limited.^13)^ Here, we present a young woman with pancreatic-type adenocarcinoma arising from HP in the Lt. She was successfully treated with multidisciplinary management based on pancreatic cancer protocols. This case provides practical information on diagnosis and treatment for this rare condition.

**Table 1:** Reported cases of esophageal HP and our case

Case	Author	Year	Sex	Age	Symptoms	Location	Treatment	Pathology	Prognosis
1	Guillou et al.^7)^	1994	M	60	Dysphagia and epigastric pain	Lt	Tumor resection + proximal gastrectomy	Adenocarcinoma	NR
2	Roshe et al.^8)^	1996	M	45	Progressive dysphagia	Lt	Thoracoabdominal esophagogastrectomy	Anaplastic cancer	NR
3	Halkic et al.^9)^	2001	M	60	Epigastric pain	EGJ	Left thoracotomy with tumor resection, proximal gastrectomy, and esophagogastric anastomosis	Adenocarcinoma	Death 3 months after surgery
4	Crighton et al.^10)^	2012	F	58	Dysphagia	EGJ	Minimally invasive Ivor Lewis esophagectomy	IPMN	Alive and well at 3 months
5	Ulrych et al.^11)^	2015	NR	NR	Dysphagia	Lt	Local esophageal resection	PanIN	Alive and well at 3 months
6	Yang et al.^12)^	2022	M	60	Epigastric discomfort	Mt	Minimally invasive McKeown esophagectomy	Adenocarcinoma	Recurred at 37 months; died 20 months later
7	Our case	2025	F	33	Upper abdominal pain	Lt	Tumor resection → GnP → esophagectomy	Adenocarcinoma	No recurrence at 12 months

This table shows case ID, sex, age, symptoms, location (EGJ/Lt/Mt), treatment, pathology, and prognosis.

EGJ, esophagogastric junction; GnP, gemcitabine plus nab-paclitaxel; HP, heterotopic pancreas; IPMN, intraductal papillary mucinous neoplasm; Lt, lower thoracic esophagus; Mt, middle thoracic esophagus; NR, not reported; PanIN, pancreatic intraepithelial neoplasia

## CASE PRESENTATION

**Fig. 1:**
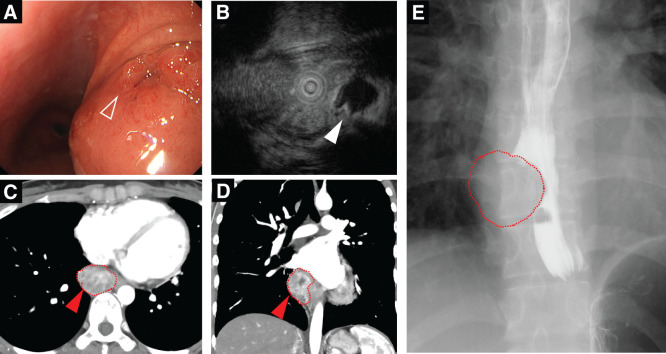
(**A**) Upper gastrointestinal endoscopy shows a submucosal tumor with a small papillary protrusion in the Lt (open arrowhead), covered by heterotopic gastric-type mucosa. (**B**) Endoscopic ultrasonography demonstrates a well-circumscribed, homogeneous hypoechoic mass (arrowhead) arising from the esophageal wall. (**C**, **D**) Contrast-enhanced CT (axial view in **C**; coronal view in **D**) reveals a well-defined extraluminal mass along the right wall of the Lt (red arrowheads). The dotted lines outline the extent of the lesion, which shows heterogeneous enhancement with focal internal low-attenuation areas. (**E**) Barium esophagography demonstrates a smooth-contoured elevated lesion along the right wall of the Lt (dotted outline). Lt, lower thoracic esophagus

**Fig. 2:**
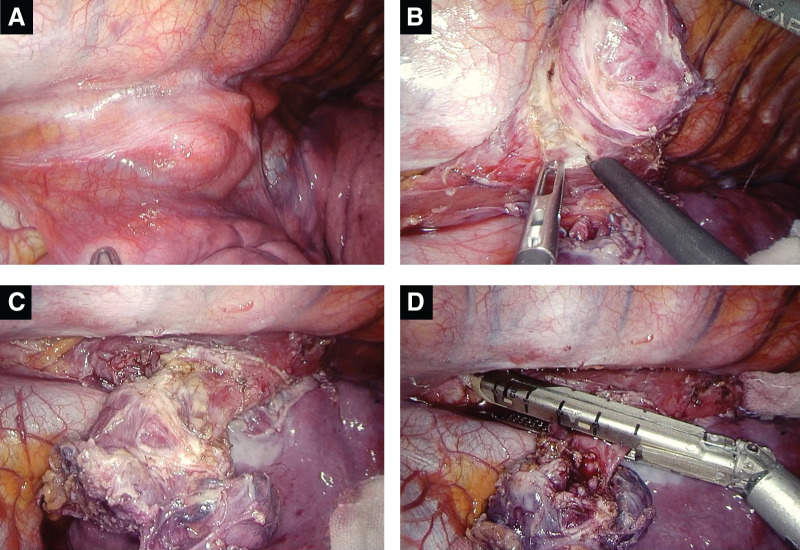
Intraoperative findings during thoracoscopic tumor resection (**A**) Thoracoscopic view showing a well-demarcated tumor protruding from the esophageal wall in the middle to lower mediastinum. The tumor was covered by mediastinal pleura and showed no macroscopic invasion into adjacent organs. (**B**) After incision of the mediastinal pleura, the tumor was carefully dissected from the surrounding tissues. Although broadly attached to the esophageal muscular layer, the lesion remained well circumscribed. (**C**) The tumor base was progressively mobilized by meticulous dissection of the esophageal muscular layer, narrowing the tumor neck while preserving the esophageal lumen. (**D**) Under transoral endoscopic guidance to confirm luminal patency and determine the optimal resection line, the tumor base was transected using a surgical stapler. The muscular defect was subsequently reinforced with continuous sutures.

**Fig. 3:**
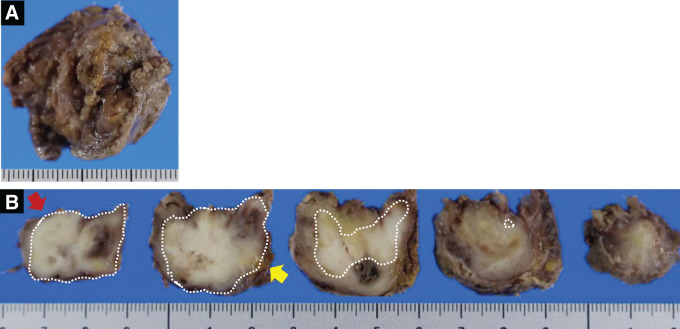
Macroscopic and histopathological findings. (**A**) Gross appearance of the resected specimen viewed from the external surface, with orientation indicated (cranial side to the right, caudal side to the left). (**B**) Serial sections of the resected specimen (cranial side to the right, caudal side to the left). The white dotted lines outline the extent of the tumor. The red arrow indicates adenocarcinoma extending beyond the muscularis propria into the adventitia and exposed at the dissected adventitial surface, indicating a positive dissected margin (RM1). The yellow arrow indicates direct invasion into an adjacent lymph node.

**Fig. 4:**
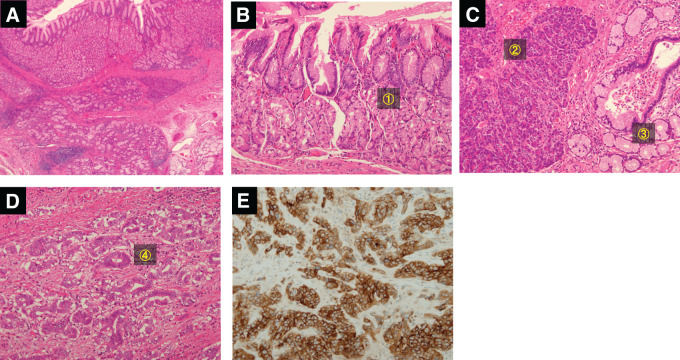
Histopathological and immunohistochemical findings. (**A**) Low-power histological view showing the overall architecture of the lesion, composed of heterotopic gastric and pancreatic tissues (hematoxylin and eosin staining). (**B**) Higher magnification of the superficial layer showing gastric-type mucosa with foveolar epithelium and fundic glands, consistent with heterotopic gastric mucosa (①) (hematoxylin and eosin staining). (**C**) Higher magnification of the deep layer demonstrating ectopic pancreatic tissue composed of pancreatic acini (②) and pancreatic ducts (③) (hematoxylin and eosin staining). (**D**) High-power view showing infiltrating adenocarcinoma with irregular glandular structures (④) arising adjacent to the ectopic pancreatic tissue (hematoxylin and eosin staining). (**E**) Immunohistochemical staining demonstrating diffuse membranous positivity for MUC1 in the adenocarcinoma cells, supporting pancreatic-type differentiation.

**Fig. 5:**
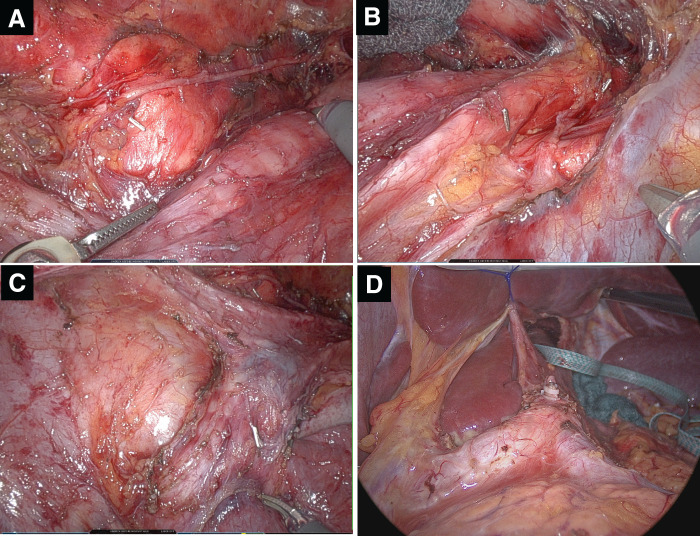
Intraoperative views during robot-assisted subtotal esophagectomy with 2-field lymphadenectomy. (**A**) Left upper mediastinal lymphadenectomy. (**B**) Right upper mediastinal lymphadenectomy. (**C**) Middle mediastinal (subcarinal) dissection around the tracheal bifurcation. (**D**) Abdominal lymph node dissection.

**Fig. 6:**
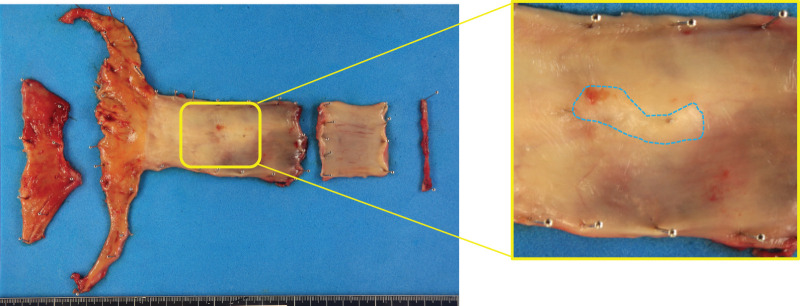
Gross specimen after esophagectomy. Left: Resected esophagus opened longitudinally and pinned. Right (magnified): The previous resection site shows scar formation without residual tumor (outlined).

A 32-year-old woman presented to Japanese Red Cross Okayama Hospital with persistent epigastric pain accompanied by severe right-sided posterior thoracic pain. The pain was intermittent but sharp and stabbing in nature, reaching a maximum intensity of 7–8/10 on the Numeric Rating Scale. Notably, the pain was strongly positional; she was unable to tolerate the supine position because of symptom exacerbation and could rest only in the prone position. These symptoms progressively worsened and markedly impaired her daily activities. She had no notable medical or family history. Physical examination and routine laboratory tests, including inflammatory markers and tumor markers, were unremarkable. Upper gastrointestinal endoscopy revealed ectopic gastric mucosa with a papillary elevation in the Lt (**Fig. 1A**). EUS demonstrated a homogeneous hypoechoic tumor arising from the esophageal wall (**Fig. 1B**). Contrast-enhanced CT showed a well-defined mass with heterogeneous enhancement in the Lt, without evidence of distant metastasis or lymphadenopathy (**Fig. 1C** and **1D**). Esophagography revealed a smooth, elevated lesion measuring approximately 30 mm on the right wall of the Lt (**Fig. 1E**). Based on these findings, because a potentially malignant esophageal lesion could not be excluded, further diagnostic evaluation with EUS-FNA and referral to an academic center for a second opinion were initially recommended. However, the patient declined both options because of intractable pain and strongly requested immediate surgical intervention for symptom relief. Given the severity of her symptoms, the positional nature of the pain, and the absence of radiological findings suggestive of advanced malignancy or metastasis, surgical intervention was selected as a diagnostic and therapeutic procedure after thorough informed consent. According to the operative record from Japanese Red Cross Okayama Hospital, thoracoscopic tumor resection was performed in the left semi-prone position under artificial pneumothorax using a 4-port approach. Intraoperatively, a well-demarcated tumor was identified in the middle to lower mediastinum, protruding from the esophageal wall and covered by the mediastinal pleura (**Fig. 2A**). After incision of the mediastinal pleura, the tumor was carefully mobilized from the adjacent esophagus as much as possible. The muscular layer at the broad-based origin of the tumor was meticulously dissected to narrow the tumor neck and facilitate safe mobilization of the lesion (**Fig. 2B** and **2C**). Under transoral endoscopic guidance to confirm luminal patency and determine the optimal resection line, the tumor base was transected using a surgical stapler (**Fig. 2D**). Finally, the esophageal muscular layer at the resection site was reinforced with continuous sutures to prevent postoperative complications. The procedure was completed without intraoperative complications. Gross examination of the thoracoscopically resected specimen revealed a solid, whitish tumor with an irregular cut surface, suggesting an infiltrative growth pattern (**Fig. 3A** and **3B**). Histopathological examination of the resected specimen revealed HP of Heinrich type I, consisting of pancreatic acini, ducts, and islets, with an associated adenocarcinoma component (**Fig. 4**). Serial section analysis demonstrated that the adenocarcinoma extended beyond the muscularis propria into the adventitia and was exposed at the dissected surface, indicating a positive dissected margin (RM1). In addition, direct invasion into an adjacent lymph node was observed (**Fig. 3B**). Further histological analysis demonstrated that the lesion comprised both gastric and pancreatic elements (**Fig. 4**). The superficial layer showed gastric-type mucosa with foveolar epithelium and fundic glands, consistent with heterotopic gastric mucosa (**Fig. 4A** and **4B**). In contrast, the deeper layer contained ectopic pancreatic tissue composed of pancreatic ducts, acinar cells, and scattered islets (**Fig. 4C**). Within this lesion, infiltrating adenocarcinoma with irregular glandular structures was observed in close association with the ectopic pancreatic tissue (**Fig. 4D**). To determine the origin of the carcinoma, immunohistochemical analysis was performed. The tumor cells showed diffuse positivity for MUC1 (**Fig. 4E**), focal positivity for MUC5AC (**Supplementary Fig. 1A**), and were negative for MUC2, MUC6 (**Supplementary Fig. 1B** and **1C**), BCL10, and trypsin/trypsinogen. Based on this immunophenotypic profile, the adenocarcinoma was diagnosed as arising from heterotopic pancreatic tissue rather than ectopic gastric mucosa (**Table 2**). The patient was subsequently referred to our hospital for additional treatment. Because of the positive dissected margin (RM1) and direct invasion into an adjacent lymph node, the patient was considered at high risk for residual disease and systemic recurrence. Therefore, adjuvant chemotherapy with gemcitabine (1000 mg/m^2^) and nab-paclitaxel (125 mg/m^2^) was administered for 3 cycles. Preoperative reassessment with contrast-enhanced CT and PET-CT revealed no evidence of recurrence or distant metastasis. Seven months after the initial surgery, robot-assisted subtotal esophagectomy with 2-field lymphadenectomy was performed (**Fig. 5**). Postoperative pathological examination demonstrated fibrotic scar tissue without residual carcinoma (**Fig. 6**), and no lymph node metastasis was identified. The patient declined further adjuvant therapy and remains free of recurrence 12 months after the second surgery.

**Table 2:** Expression sites of lineage-associated markers

Core protein	Main sites of expression
MUC1	Pancreatic ductal cells, intercalated ducts, mammary gland
MUC2	Small intestine, large intestine, airway
MUC5AC	Gastric foveolar epithelial cells
MUC6	Pyloric gland, salivary gland, gastric chief cells duodenal Brunner’s glands, esophageal submucosal glands
BCL10 trypsin/trypsinogen	Pancreatic acinar cells

This table lists each core protein and its predominant tissues of expression used for immunophenotypic interpretation (gastric/intestinal/pancreatobiliary/acinar differentiation).

## DISCUSSION

HP is pancreatic tissue located at an abnormal site without anatomical or vascular continuity with the normal pancreas, with a reported prevalence of 0.5%–13.7% in the general population.^5)^ Most cases are asymptomatic and incidentally detected, and the stomach, duodenum, and jejunum are the most common locations. Esophageal HP is exceedingly rare, with only 17 cases reported to date, and typical symptoms include epigastric pain, dysphagia, and gastrointestinal bleeding.^14)^ Our literature review identified only 5 cases of malignant transformation arising from esophageal HP. When premalignant lesions such as IPMN and PanIN were included, a total of 7 cases have been reported (**Table 1**). Most patients presented with dysphagia or epigastric pain, and surgical resection was the primary treatment in all reported malignant cases. Reported surgical procedures ranged from local tumor resection to esophagectomy, depending on tumor extent and preoperative diagnostic suspicion. Although the number of reported cases remains extremely limited, several patients achieved favorable outcomes after complete surgical resection, suggesting that early diagnosis and multidisciplinary treatment may improve prognosis. According to a recent review, malignant transformation of HP is rare and difficult to diagnose preoperatively; of 54 reported malignant HP cases, most originated in the stomach and were adenocarcinomas, showing relatively favorable outcomes compared with conventional pancreatic cancer.^15)^ With respect to preoperative assessment, EUS-FNA is a useful diagnostic modality, but its sensitivity is limited to approximately 50%–80%.^16)^ In the present case, severe positional thoracic pain made diagnostic biopsy difficult, and surgical resection was chosen for symptom relief in accordance with the patient’s strong preference after informed consent. Histopathological examination revealed a diverticulum-like cavity containing food debris and seed-like foreign material, accompanied by suppurative inflammatory changes (**Supplementary Fig. 2**), which was considered a possible cause of the severe pain. Notably, the patient’s symptoms completely resolved after tumor resection. At the time of the initial surgery, the lesion was regarded as a submucosal tumor with no pathological diagnosis of malignancy. While esophagectomy with reconstruction was considered, the procedure was judged to be overly invasive given the unconfirmed malignant status. Consequently, thoracoscopic tumor resection was selected as a less invasive approach for both diagnostic and therapeutic purposes. Had EUS-FNA provided a definitive diagnosis of adenocarcinoma arising from HP, neoadjuvant chemotherapy followed by subtotal esophagectomy with lymphadenectomy might have been the optimal initial strategy. On histopathology after the first surgery, the site of origin of the adenocarcinoma (ectopic gastric mucosa vs. HP) was the key determinant for the subsequent treatment strategy. To clarify this, we performed immunohistochemistry, which showed MUC1 positivity,^17)^ with focal MUC5AC positivity, and negativity for MUC2, MUC6, BCL10, and trypsin/trypsinogen.^18–20)^ This expression profile supported a diagnosis of adenocarcinoma arising from HP.

There is no established standard therapy for carcinoma arising from HP. Surgical resection is generally considered first-line for resectable disease, while multidisciplinary treatment including chemotherapy and/or radiotherapy is considered for advanced cases. In our patient, histopathological examination after the initial surgery demonstrated pancreatic-type adenocarcinoma with a positive dissected margin (RM1) and direct invasion into an adjacent lymph node. Because the biological behavior of this tumor was considered similar to pancreatic adenocarcinoma, systemic chemotherapy with GnP was administered according to pancreatic cancer treatment protocols.^21)^ The aims of chemotherapy before the second surgery were to control potential micrometastatic disease, reduce the risk of local recurrence, and confirm the absence of progressive systemic disease prior to radical surgery. After confirming the absence of recurrence or distant metastasis, robot-assisted subtotal esophagectomy was performed with curative intent, achieving a pathological complete response.

Although outcome data remain limited, the prognosis of HP-associated carcinoma may be relatively favorable compared with conventional pancreatic adenocarcinoma, possibly owing to earlier detection and resectability. Accumulation of additional cases is needed to improve diagnostic accuracy and to establish standardized treatment protocols. In addition, careful long-term follow-up is essential because the biological behavior and recurrence pattern of carcinoma arising from esophageal HP remain poorly understood.

## CONCLUSIONS

This report highlights successful multidisciplinary management of esophageal HP carcinoma modeled on pancreatic cancer protocols, achieving complete pathological remission and favorable outcomes. Due to its malignant potential, careful preoperative assessment and individualized treatment planning are crucial.

## SUPPLEMENTARY MATERIALS

Supplementary Figure 1 Immunohistochemical findings of mucin expression in the tumor. (**A**) MUC5AC immunostaining demonstrates focal cytoplasmic positivity in the tumor cells. (**B**) MUC2 immunostaining shows no expression in the tumor cells. (**C**) MUC6 immunostaining is negative in the tumor cells.

Supplementary Figure 2 Histopathological findings potentially associated with severe pain. (**A**) Suppurative inflammatory changes with abundant inflammatory cell infiltration within the lesion. (**B**) Food debris and seed-like foreign material within a diverticulum-like cavity, accompanied by inflammatory changes.

## DECLARATIONS

### Availability of data and materials

All relevant datasets supporting the conclusions of this article are included within the manuscript.
