# Supplementary figures and images for: Multidisciplinary Treatment for Advanced Pancreatic Adenocarcinoma Arising from Esophageal Heterotopic Pancreas in a Young Adult: A Case Report
Source: Surg Case Rep. 2026 Jun 27;12(1):26-0088. doi: 10.70352/scrj.cr.26-0088 (PMC13318428; doi:10.70352/scrj.cr.26-0088)

Supplementary Figure 1.

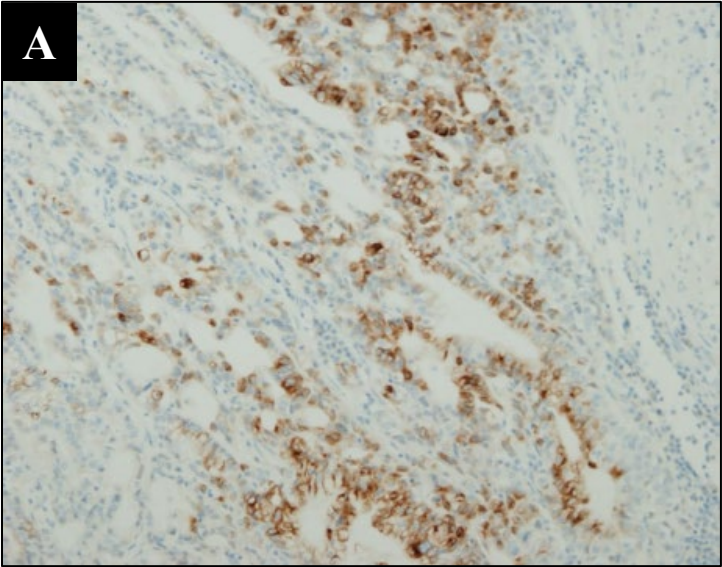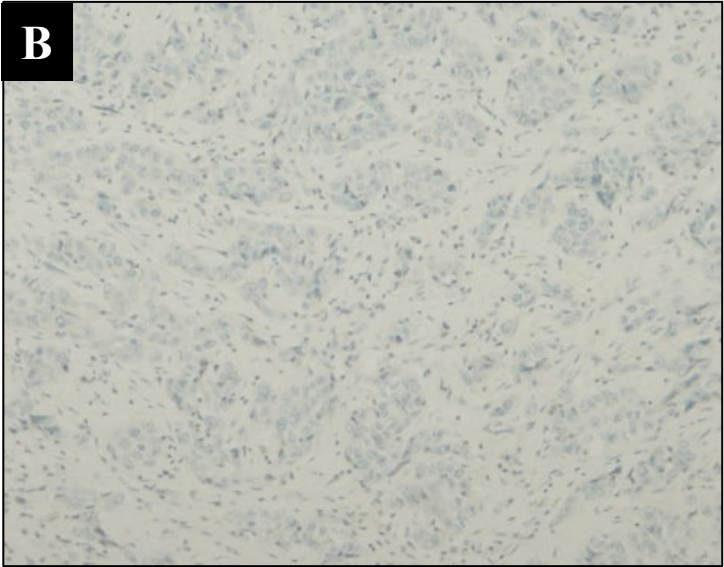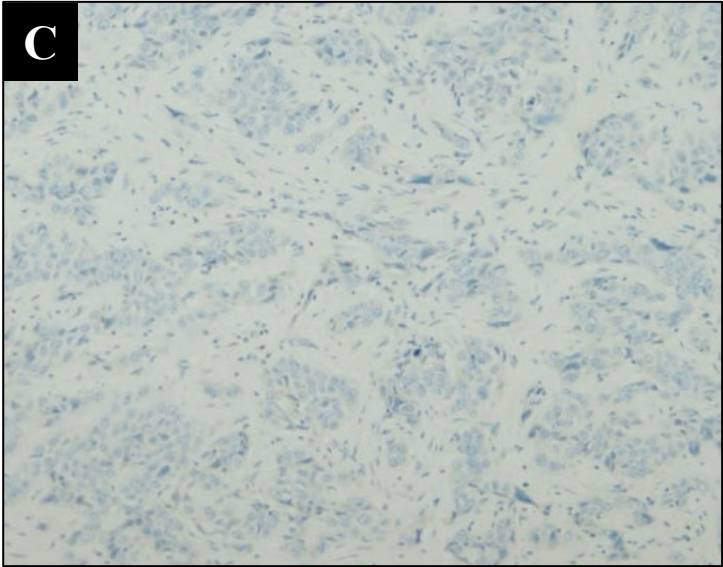

Supplementary Figure 2.

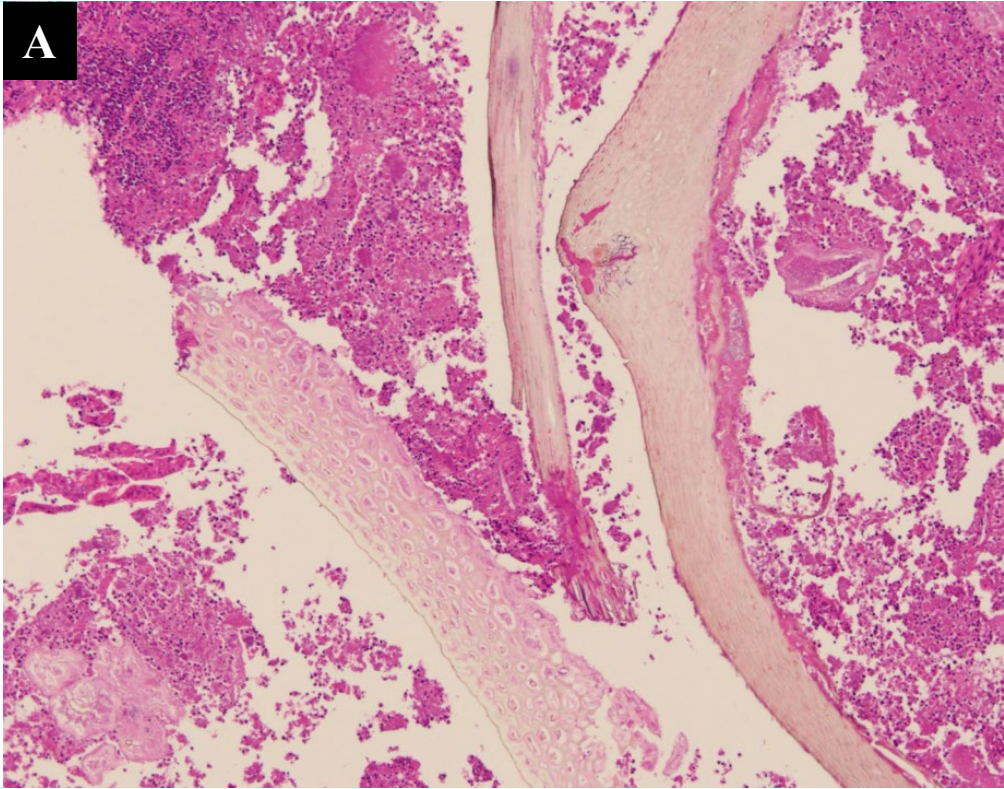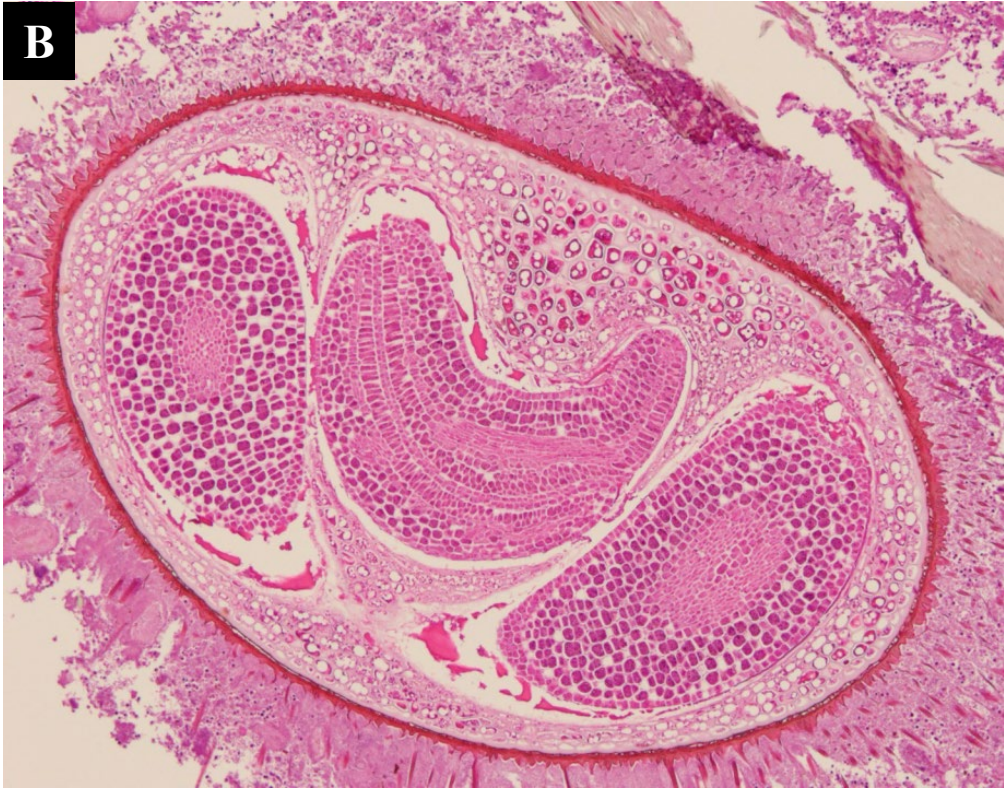

Supplement: Supplementary Figure 1 — Immunohistochemical findings of mucin expression in the tumor. (A) MUC5AC immunostaining demonstrates focal cytoplasmic positivity in the tumor cells. (B) MUC2 immunostaining shows no expression in the tumor cells. (C) MUC6 immunostaining is negative in the tumor cells. [file scr-12-01-26-0088-s001.pdf]
